# Longitudinal association between serum uric acid levels and multiterritorial atherosclerosis

**DOI:** 10.1111/jcmm.14337

**Published:** 2019-06-26

**Authors:** Meiyue Song, Na Li, Yan Yao, Kaile Wang, Jichun Yang, Qinghua Cui, Bin Geng, Jianxin Chen, Youxin Wang, Wenli Cheng, Yong Zhou

**Affiliations:** ^1^ Beijing University of Chinese Medicine and China‐Japan Friendship Hospital Beijing China; ^2^ Department of Health Care China‐Japan Friendship Hospital, Ministry of Health Beijing China; ^3^ Department of Cardiology Beijing Anzhen Hospital, Capital Medical University Beijing China; ^4^ Department of Physiology and Pathophysiology Peking University Health Science Center Beijing China; ^5^ Hypertension Center, Fuwai Hospital, Chinese Academy of Medical Sciences and Peking Union Medical College State Key Laboratory of Cardiovascular Disease Beijing China; ^6^ Beijing Key Laboratory of Clinical Epidemiology, School of Public Health Capital Medical University Beijing China; ^7^ Department of Hypertension Beijing Anzhen Hospital and Capital Medical University Beijing China; ^8^ Sanbo Brain Institute Sanbo Brain Hospital, Capital Medical University Beijing China

**Keywords:** Asymptomatic Polyvascular Abnormalities in Community, hyperuricaemia, multiterritorial vascular stenosis, prospective cohort study, serum uric acid

## Abstract

Multiterritorial atherosclerosis has dramatically increased annual risk of adverse cardiovascular events than atherosclerotic disease with single‐artery affected. Serum uric acid (SUA) is an important predictor of stroke and atherosclerosis; however, which is supported by few direct evidence based on cohort studies. A prospective cohort study including 2644 North Chinese adults aged ≥40 years was performed in 2010‐2012 to investigate the association between SUA and multiterritorial vascular stenosis. Hyperuricaemia was defined as SUA levels >6 and >7 mg/dL for males and females, respectively. All participants underwent twice transcranial Doppler (TCD) and bilateral carotid duplex ultrasound to evaluate intracranial artery stenosis (ICAS) and extracranial arterial stenosis (ECAS) and peripheral arterial disease (PAD) was determined by ankle‐brachial index (ABI) on January 2010 and January 2012 based on regular health check‐ups. The cumulative incidence of vascular stenosis was significantly higher in subjects with hyperuricaemia than in those without hyperuricaemia (54.1% vs. 34.7%, *P* < 0.001). The adjusted odds ratios (ORs) with 95% confidence intervals (CIs) for new on‐set vascular stenosis due to hyperuricaemia and a 1‐mg/dL change in SUA level were 1.75 (1.32‐2.31) and 1.29 (1.21‐1.38), respectively. Furthermore, in the gender‐stratified analysis, the association between SUA levels and ICAS was statistically significant in males (OR: 2.02; 95% CI: 1.18‐3.46), but not females (OR: 0.85, 95% CI: 0.41‐1.76, *P* for interaction: 0.026).

## 1 INTRODUCTION

Atherosclerosis is the leading cause of cardiovascular death and disability worldwide,^1^ and the consequences of atherosclerosis as a systemic disease with manifestations in more than one arterial territory have been a research focus.^2^ The annual incidence of cardiovascular events is markedly higher in patients with multiple established atherosclerotic arterial disease regions than in patients with atherosclerosis affecting only one arterial territory.^3^ During the last decade, interest has increased in multifocal atherosclerotic disease, the prevalence of which is approximately 16% to 40%.^4^, ^5^, ^6^ Calcification, plaques and vascular stiffness across multiple arteries are often used as surrogate markers of the presence of polyvascular atherosclerosis.^4^, ^5^, ^7^

Serum uric acid (SUA), the end‐product of purine nucleotides metabolism, are widely recognized as a risk factor for atherosclerosis.^8^ However, the independence of this association remains controversial.^9^ Previous studies have focused intensively on patients with single‐territory disease^10^, ^11^ or patients with fewer atherosclerosis‐affected territories,^12^ which may make it difficult to comprehensively evaluate the association between uric acid levels and atherosclerotic burden. Furthermore, a recent meta‐analysis demonstrated that baseline SUA levels are an independent predictor of future cardiovascular mortality,^13^ indicating a clear need to evaluate this manifestation in early‐stage cardiovascular disease. Few studies have provided direct evidence that SUA levels are associated with multiple arterial stenosis, one of the top contributors to adverse cardiovascular events. Additionally, previous evidence predominantly consists of data obtained from cross‐sectional studies. In light of these previous studies, we have suggested that there is an independent, longitudinal relationship between elevated SUA levels and multifocal vascular stenosis. We tested this hypothesis in the prospective, community‐based Asymptomatic Polyvascular Atherosclerosis Community cohort study (APAC).

## 2 METHODS

### 2.1 Study design and population

The study subjects were participants in the APAC study, which aimed to investigate the epidemiology of asymptomatic polyvascular abnormalities, including asymptomatic intracranial artery stenosis (ICAS), extracranial artery stenosis (ECAS) and peripheral artery disease (PAD), in Chinese adults.^6^ The inclusion criteria of the study are as follows: (1) ≥40 years old; (2) complete basic information available. The exclusion criteria including: (1) suffering intracranial artery stenosis (ICAS), extracranial artery stenosis (ECAS) and peripheral artery disease (PAD); (2) estimated glomerular filtration rate (eGFR) ≤60 mL/min/1.73 m^2^; (3) with the history of stroke, transient ischaemic attack and neurological deficits, myocardial infarction, coronary heart disease.

Two years later, all the participants were scheduled to undergo the second assessment of ICAS, ECAS and PAD. A total of 2644 participants (51.7% males) with twice available TCD, bilateral carotid duplex ultrasound and the ABI were included in the final analyses.

The study was conducted in accordance with the guidelines of the Helsinki Declaration and approved by the Kailuan General Hospital and the Beijing Tiantan Hospital Ethics Committee (2010‐014‐01). Signed informed consent was obtained from all participants.

### 2.2 Determination of serum uric acid levels and definition of hyperuricaemia

Blood samples were collected from the antecubital region in the morning under fasting conditions (at least 12 hours) and after the patients had been in a sitting position for 15 minutes. All blood samples were stored in vacuum tubes containing EDTA (Ethylene Diamine Tetraacetic Acid) and processed and analysed using an auto‐analyser (Hitachi 747; Hitachi, Tokyo, Japan) at the central laboratory of the Kailuan General Hospital. Hyperuricaemia was defined as >7.0 mg/dL for men and >6.0 mg/dL for women.^14^, ^15^

### 2.3 Assessment of multiterritorial atherosclerosis

Vascular stenosis was defined as the presence of any stenosis in the intracranial, extracranial and peripheral arteries.^6^

Assessment of intracranial artery stenosis (ICAS) was assessed by peak systolic flow velocity measurements via transcranial Doppler (TCD) according to published criteria.^16^ Vascular stenosis was defined as follows: (1) >140 cm per second (cm/s) in the middle cerebral artery; (2) >120 cm/s in the anterior cerebral artery; (3)>100 cm/s in the posterior cerebral artery and vertebra‐basilar artery and (4) >120 cm/s in the siphon of the internal carotid artery. Two experienced neurologists who were blinded to the baseline information of the patients rigorously complied with the published standardized protocol for performing TCD with portable machines (Nicolet/EME Company, Germany). If the two operators disagreed, a third qualified senior arbiter reviewed the case and a final decision was achieved by consensus.

Extracranial artery stenosis (ECAS) was defined as the presence of a common or extracranial internal carotid artery stenosis or extracranial vertebral artery stenosis. All participants underwent a bilateral carotid duplex ultrasound (Philips iU‐22 ultrasound system, Philips Medical Systems, Bothell, WA) in the supine position of structures including the common carotid artery, internal carotid artery, external carotid artery, vertebral artery and subclavian artery. Both sides of the carotid arteries were assessed for the presence of ECAS (≥50%), which was graded based on recommendations from the Society of Radiologists in Ultrasound Consensus Conference.^17^

Peripheral artery disease (PAD) was assessed by the ankle brachial index (ABI), which was calculated as the ratio of ankle to brachial systolic blood pressure. The systolic pressures of the bilateral dorsalis pedis artery, posterior tibial artery and brachial artery were recorded by a portable Doppler device (Hokanson MD6Doppler with MD6VR Chart Recorder; Bellevue, WA) after the patient had completed a had rested for 10 minutes in a supine position. The ABI was calculated bilaterally by dividing the higher value between the dorsalis pedis artery and the posterior tibial artery systolic pressures by the higher value between the bilateral brachial artery systolic pressures. The lower ABI value was then analysed. An ABI ≤ 0.90 indicated the presence of abnormalities.^18^

### 2.4 Covariates

Data related to demographic variables, diseases history and life style were collected by qualified investigators using standardized questionnaires, and biochemical indicators were assessed at Kailuan General Hospital (Hitachi 747; Hitachi, Tokyo, Japan), as previously described.^19^ The covariates included age, sex, body mass index (BMI), education level, monthly per capita income, smoking status, alcohol consumption, physical activity, hypertension, diabetes mellitus, hyperlipidaemia, total cholesterol (TC) levels, triglyceride (TG) levels, high‐density lipoprotein TC (HDL‐C) levels, low density lipoprotein TC (LDL‐C) levels C‐reactive protein (CRP), serum albumin (ALB) and eGFR.

Physical activity was assessed based on lifestyle behaviours by combining occupational and discretionary physical activities (including the following three categories: inactive, moderately active and vigorously active^20^). Hypertension was defined as systolic blood pressure ≥140 mmHg, diastolic blood pressure ≥90 mm Hg, history of hypertension, or receiving antihypertensive medication.^21^ Diabetes mellitus was defined as a fasting plasma glucose level ≥126 mg/dL, a self‐reported history, or current treatment with insulin or oral hypoglycaemic agents.^22^ Hyperlipidaemia was defined as a total TC level of ≥220 mg/dL, TG levels of ≥150 mg/dL, LDL‐C levels ≥160 mg/dL, previous or current use of lipid‐lowering medicine.^23^

### 2.5 Statistical analysis

Continuous variables are present as the mean ± standard derivation, and categorical variables are present as percentages. Differences in continuous variables between SUA categories were tested evaluated the *t* test or Kruskal‐Wallis test, depending on whether the data were or were not normally distributed, respectively. The chi‐squared test (or Fisher's exact test for frequencies <5) was performed for categorical variables.

A multivariable logistic regression analysis was used to determine the association between elevated SUA levels (hyperuricaemia and per 1 mg/dL elevated) and new multiterritorial vascular stenosis by calculating the odd ratios (ORs) with 95% confidence intervals (CIs) after adjusting for age, sex, BMI, income, education level, smoking, alcohol consumption, physical activity, hypertension, diabetes mellitus, hyperlipidaemia, CRP, ALB, and eGFR. Moreover, restricted cubic spline (RCS) logistic regression models were used to investigate any non‐linear effects of SUA on vascular stenosis risk by treating them as continuous covariates.

Chronic kidney disease (CKD) is one of strongest coronary risk factors. Therefore, the multivariable logistic regression analysis was performed by eGFR‐stratification. Also, to further control the effect of this confounding factor, a propensity score‐matched analysis was conducted between subjects suffering from new vascular stenosis and those not. Propensity scores were developed accounting for variables including age and eGFR by logistic regression analysis. After matching, the multivariable logistic regression analysis was also conducted as mentioned above.

All the multivariable logistic regression analyses were also performed stratified by gender due to gender difference in SUA levels.

All statistical tests were two‐sided, and the level of significance was set at *P* < 0.05. All statistical analyses were performed using SAS software (version 9.3; SAS Institute Inc, Cary, NC).

## 3 RESULTS

### 3.1 Baseline characteristics

**Figure 1:**
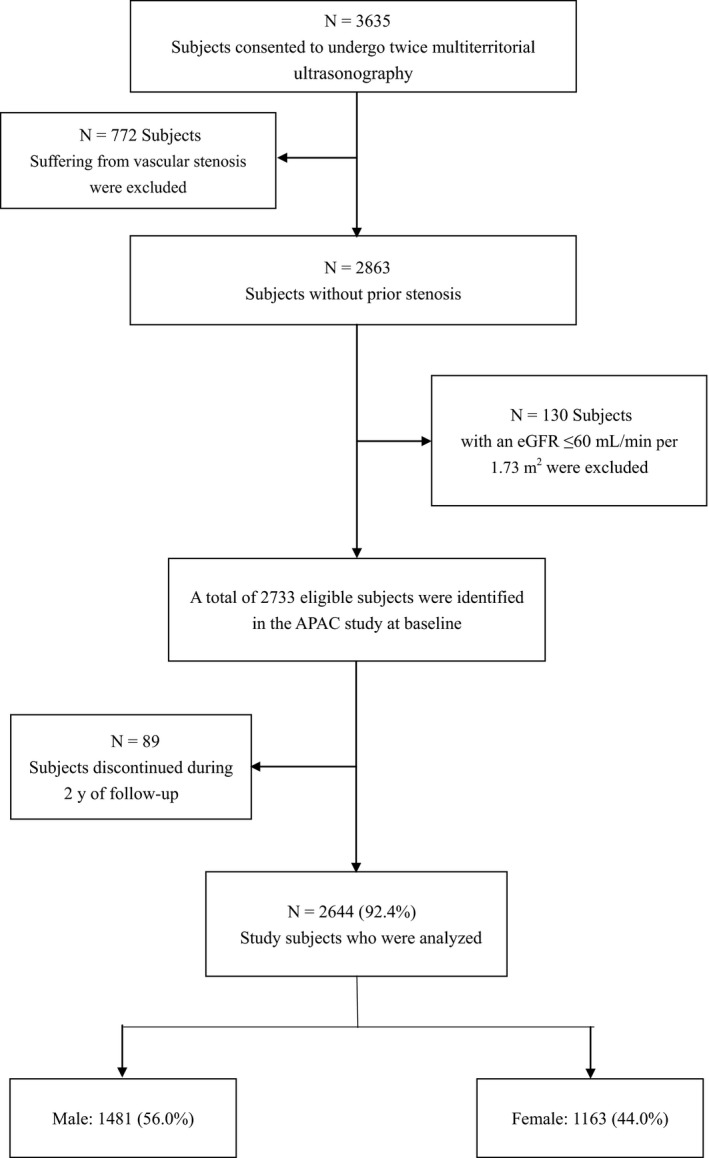
The flow chart

Of the 3635 participants, 2644 subjects were available for the final analyses; 772 subjects suffering from the vascular stenosis and 130 subjects with an eGFR *≤*60 mL/min/1.73 m^2^ and 89 subjects who did not complete the study were excluded. (Figure 1).

The baseline characteristics of the participants are summarized in Table 1. The average age of the participants was 53.3 years old, and 56% were males. The prevalence of hyperuricaemia was 10.2% (270/2644), and this prevalence was higher in men than in women (11.9% vs 8.0%, *P* < 0.001). Hyperuricaemic participants had higher proportions of hypertension and hyperlipidaemia, and a higher BMI and lower eGFR than non‐hyperuricaemics.

**Table 1:** Baseline characteristics of participants with or without hyperuricaemia

Characteristic	Total (n = 2644）	Hyperuricaemia (n = 270)	Non‐hyperuricaemia (n = 2374)	*P* value
Male sex, n (%)	1481 (56.0)	176 (65.2)	1305 (55.0)	0.001
BMI (SD), kg/m^2^	24.9 ± 3.2	26.3 ± 3.4	24.7 ± 3.1	<0.001
Education level				
Primary school or low, n (%)	243 (9.2)	24 (8.9)	219 (9.2)	0.094
Middle or high school, n (%)	1123 (42.5)	99 (36.7)	1024 (43.1)	
College or above, n (%)	1278 (48.3)	147 (54.4)	1131 (47.6)	
Income				
≤500, n (%)	27 (1.0)	3 (1.1)	24 (1.0)	<0.001
500‐1000, n (%)	510 (19.3)	33 (12.2)	477 (20.1)	
1000‐3000, n (%)	1789 (67.7)	178 (65.9)	1611 (67.9)	
>3000, n (%)	318 (12.0)	56 (20.7)	262 (11.0)	
Alcohol consumption				
Light, n (%)	479 (18.1)	63 (23.3)	416 (17.5)	<0.001
Moderate, n (%)	310 (11.7)	46 (17.0)	264 (11.1)	
Heavy, n (%)	68 (2.6)	14 (5.2)	54 (2.3)	
Smoking				
Never, n (%)	1691 (64.0)	154 (57.0)	1537 (64.7)	0.026
Once, n (%)	129 (4.9)	19 (7.0)	110 (4.6)	
Currently, n (%)	824 (31.2)	97 (35.9)	727 (30.6)	
Physical activity				
Inactive, n (%)	1043 (39.4)	87 (32.2)	956 (40.3)	0.037
Moderately active, n (%)	696 (26.3)	79 (29.3)	617 (26.0)	
Vigorously active, n (%)	905 (34.2)	104 (38.5)	801 (33.7)	
Hypertension, n (%)	1125 (42.5)	166 (61.5)	959 (40.4)	<0.001
Diabetes mellitus, n (%)	257 (9.7)	21 (7.8)	236 (9.9)	0.256
Hyperlipidaemia, n (%)	1268 (48.0)	196 (72.6)	1072 (45.2)	<0.001
TC (SD), mmol/L	1.5 ± 0.7	1.7 ± 0.8	1.5 ± 0.7	0.001
TG (SD), mmol/L	1.7 ± 1.5	2.5 ± 2.0	1.6 ± 1.4	<0.001
HDL‐C (SD), mmol/L	1.6 ± 0.42	1.5 ± 0.4	1.6 ± 0.4	<0.001
LDL‐C (SD), mmol/L	2.6 ± 0.8	2.6 ± 0.8	2.6 ± 0.8	0.422
CRP (SD), mg/L	1.9 ± 3.3	2.7 ± 3.0	1.8 ± 3.4	<0.001
ALB (SD), g/L	48.8 ± 9.2	48.5 ± 9.8	48.9 ± 9.1	0.504
SUA (SD), mg/dL	4.9 ± 1.5	7.7 ± 1.1	4.5 ± 1.2	<0.001
eGFR (SD), mL/min per 1.73 m^2^	100.0 ± 22.4	96.9 ± 19.2	100.4 ± 22.7	0.015

Note

Hyperuricaemia was defined as SUA ≥7.0 and ≥6.0 mg/dL in men and women, respectively. Abbreviations: ALB, serum albumin; BMI, body mass index; HDL‐C, high‐density lipoprotein; LDL‐C, low‐density lipoprotein, CRP, C‐reactive protein; SUA serum uric acid and eGFR estimated glomerular filtration rate; TC, total cholesterol; TG, triglyceride.

### 3.2 Cumulative incidence and the distribution of vascular stenosis

**Figure 2:**
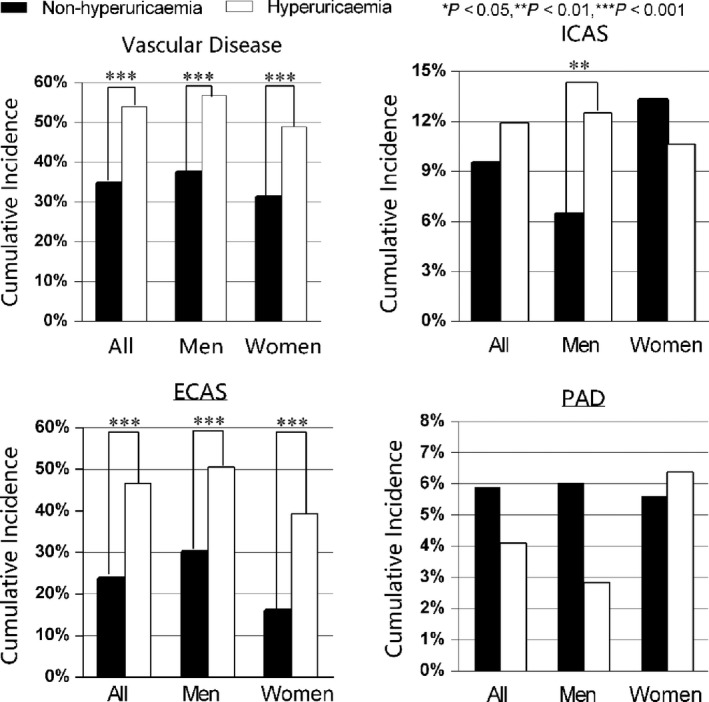
The cumulative incidence of the vascular stenosis. There was a significant difference in the cumulative incidence of stenosis between subjects with and without hyperuricaemia over 2 years of follow‐up. (***P < 0.001, **P < 0.01 by chi‐squared analysis). Vascular stenosis was defined as the presence of ICAS or ECAS at ≥1 site. ECAS, extracranial artery stenosis; ICAS, intracranial artery stenosis; PAD, peripheral artery disease; VS, vascular stenosis

Figure 2 shows the cumulative incidence of vascular stenosis and its distribution among subjects with and without hyperuricaemia. During the 2‐year follow‐up period, the incidence of vascular stenosis was 36.7% (970 subjects) (presence of stenosis at ≥1 site), among which 9.8% was ICAS, 26.1% was ECAS and 5.7% was PAD. Hyperuricaemic subjects had a significantly higher incidence of vascular stenosis (54.1% vs 34.7%, *P* < 0.001), predominantly ECAS (23.8% vs 46.7%, *P < *0.001), than normouricaemic subjects. No significant difference was found in the incidence of ICAS and PAD between participants with and without hyperuricaemia (*P* > 0.05).

### 3.3 Association between elevated SUA levels and vascular stenosis

The associations between elevated SUA levels (hyperuricaemia and per 1 mg/dL) and vascular stenosis are demonstrated in Table 2. In the univariate analyses, we found that age, gender, education level, income level, physical activity, history of hypertension, history of diabetes mellitus, history of hyperlipdaemia, CRP, SUA and eGFR were associated with the new vascular stenosis. (Table S1) In the unadjusted model, hyperuricaemic participants, as well as ones with higher SUA (per 1 mg/dL increased), had a higher risk for vascular stenosis (OR: 2.22; 95% CI: 1.72‐2.85 and OR: 1.39; 95% CI: 1.31‐1.47, respectively), especially for ECAS (OR: 2.81; 95% CI: 2.17‐3.63, but not for ICAS OR: 1.28; 95% CI: 0.86‐1.90); in the fully adjusted model, hyperuricaemia remained an independent risk factor for overall vascular stenosis (OR: 1.75; 95% CI: 1.32‐2.31) and for ECAS (OR: 2.17; 95% CI: 1.61‐2.94). However, there was no significant association between hyperuricaemia and ICAS (OR: 1.38; 95% CI: 0.91‐2.10). The SUA level per 1.0 mg/dL was associated with a 1.29 (1.21‐1.38)‐fold increased risk of overall vascular stenosis, a 1.23 (1.11‐1.36)‐fold increased risk of ICAS, and a 1.47 (1.36‐1.59)‐fold increased risk of ECAS.

**Table 2:** Odd ratios with 95% CIs for the onset of vascular stenosis due to elevated SUA

Hyperuricaemia (n = 270)	Non‐hyperuricaemia (n = 2374)	SUA risk	Odd ratios (95% CIs)			
			Unadjusted	*P* value	Adjusted	*P* value
Vascular stenosis						
146 (54.1)	824 (34.7)	Hyperuricaemia	2.22 (1.72‐2.86)	<0.001	1.75 (1.32‐2.31)	<0.001
		per SUA1 mg/dL	1.39 (1.31‐1.47)	<0.001	1.29 (1.21‐1.38)	<0.001
ICAS						
32 (11.9)	226 (9.5)	Hyperuricaemia	1.28 (0.86‐1.90)	0.222	1.38 (0.91‐2.10)	0.129
		per SUA1 mg/dL	1.10 (1.01‐1.19)	0.026	1.23 (1.11‐1.36)	<0.001
ECAS						
126 (46.7)	564 (23.8)	Hyperuricaemia	2.81 (2.17‐3.63)	<0.001	2.17 (1.61‐2.94)	<0.001
		per SUA1 mg/dL	1.61 (1.51‐1.71)	<0.001	1.47 (1.36‐1.59)	<0.001

Note

Model 1: unadjusted. Model 2: fully adjusted for age, sex, educational level, income, smoking, alcohol consumption, hypertension, hyperlipidaemia, diabetes mellitus, body mass index (BMI), C‐reactive protein (CRP), serum albumin (ALB) and estimated glomerular filtration rate (eGFR). Vascular stenosis was defined as the presence of ICAS or ECAS at ≥1 site. Abbreviations: ECAS, extracranial artery stenosis; ICAS, intracranial artery stenosis.

The incidence of abnormal ABI in the follow‐up 2 years (11/2644) is low in APAC study, consistent with previous studies with a low prevalence in middle‐aged individuals.^5^, ^24^, ^25^ Therefore, the protocol was amended to discontinue analysis of the association between ABI and vascular stenosis.

**Figure 3:**
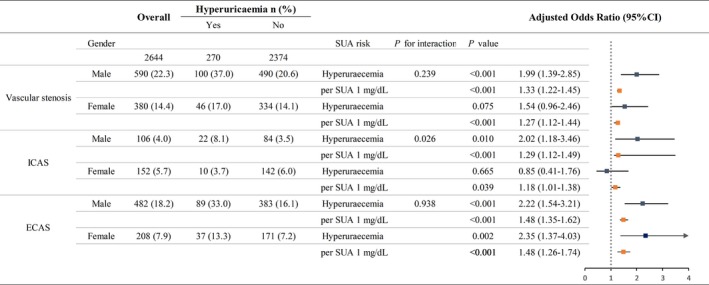
Odd ratios with 95% CI for the onset of vascular stenosis in males and females due to hyperuricaemia and a 1‐mg/dL elevation in SUA level. Fully adjusted for age, sex, educational level, income, smoking, alcohol consumption, hypertension, hyperlipidaemia, diabetes mellitus, body mass index (BMI), Creactive protein (CRP), serum albumin (ALB) and estimated glomerular filtration rate (eGFR). Vascular stenosis was defined as the presence of ICAS or ECAS at ≥1 site. ECAS, extracranial artery stenosis; ICAS, intracranial artery stenosis

**Figure 4:**
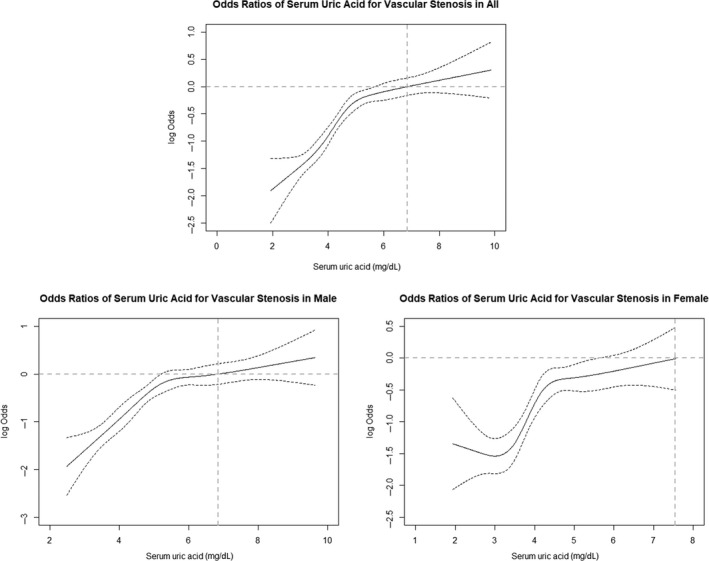
Log odds ratio and 95% confidence intervals for vascular stenosis in relation to SUA. The restricted cubic spline analysis (RCS) for SUA was fully adjusted for age, sex, educational level, income, smoking, alcohol consumption, hypertension, hyperlipidaemia, diabetes mellitus, body mass index (BMI), C‐reactive protein (CRP), serum albumin (ALB) and estimated glomerular filtration rate (eGFR). Vascular stenosis was defined as the presence of ICAS or ECAS at ≥1 site. ECAS, extracranial artery stenosis; ICAS, intracranial artery stenosis

In a gender‐stratified analyses, the association between hyperuricaemia and ICAS was statistically significant in males (OR: 2.02; 95% CI: 1.18‐3.46) but not in females (interaction *P *= 0.026). Associations of hyperuricaemia with vascular stenosis (OR: 1.99; 95% CI: 1.39‐2.85) and ECAS (OR: 2.22; 95% CI: 1.54‐3.21) were also observed in males. Only the association between hyperuricaemia and ECAS (OR: 2.35; 95% CI: 1.37‐4.03) was observed in females. Increases in the SUA level were associated with a higher risk of vascular stenosis across all target arteries in both males and females (Figure 3). These results were partly confirmed by the RCS shown in Figure 4. There is a non‐linear association between serum urate level and risk for vascular stenosis and with continuously increasing SUA, the log OR for vascular stenosis increased in all participants, indicating increased risk for vascular stenosis. The same trend was also found in both gender; however, increased risk for vascular stenosis was observed in a lower SUA range in males compared to that observed in females.

**Figure 5:**
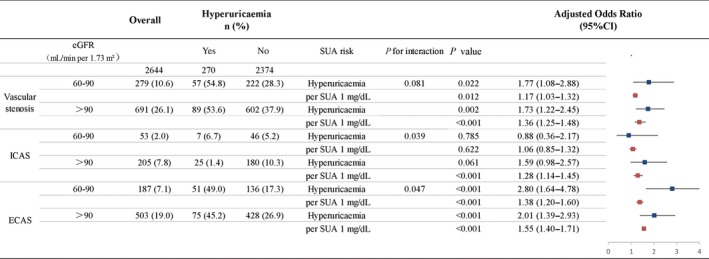
Odd ratios with 95% CI for the onset of vascular stenosis due to hyperuricaemia and a 1‐mg/dL elevation in SUA level stratified by eGFR. Fully adjusted for age, sex, educational level, income, smoking, alcohol consumption, hypertension, hyperlipidaemia, diabetes mellitus, body mass index (BMI), Creactive protein (CRP), serum albumin (ALB) and estimated glomerular filtration rate (eGFR). Vascular stenosis was defined as the presence of ICAS or ECAS at ≥1 site. ECAS, extracranial artery stenosis; ICAS, intracranial artery stenosis

In the eGFR‐stratified analyses, hyperuricaemic subjects with eGFR both ranging 60 to 90 and hinger than 90 mL/min per 1.73 m^2^ had a significantly increased risk for vascular stenosis (OR: 1.77; 95% CI: 1.08‐2.88 and OR: 1.73; 95% CI: 1.22‐2.45, respectively) and ECAS (OR: 2.80; 95% CI: 1.64‐4.78 and OR: 2.01; 95% CI: 1.39‐1.71, respectively) (Figure 5). However, the *P* for interaction were 0.081 and 0.039, respectively. For ICAS, although hyperuricaemia was not associated with a significantly increased risk in subjects with low or normal kidney function, the *P* for interaction was 0.047. Per 1.0 mg/dL SUA increasing had a significant risk for vascular stenosis and ECAS in all subjects, except for ICAS in CKD patients (OR: 1.06; 95% CI: 0.85‐1.32) (Figure 4). For the propensity‐score matched analysis, the eGFR of subjects suffering from new vascular stenosis (n = 970) and those not (n = 970) were similar after matching and the baseline characteristics of the case‐ and control‐group were presented in Table S2. After matching, Figure S1 showed that the elevated SUA (hyperuricaemia and every 1 mg/dL SUA increasing) was still significantly associated with new vascular stenosis and ECAS in both genders, but with ICAS only in males, similar to the results in Figure 3. (Line, Page).

We also examined the effect of hypouricaemia on vascular stenosis, and the results are listed in the Table S3. Only 0.45% (12/2644) female subjects suffer from hypouricaemia, and the result is consistent with our previous analysis.

## 4 DISCUSSION

In this study, we focused on the longitudinal association between elevated SUA levels and asymptomatic polyvascular stenosis in a Chinese community cohort. The main finding was that elevated SUA levels are associated with an increased risk of multifocal stenosis. Furthermore, the effect of SUA on ICAS was significant in men but not in women.

To date, few direct evidences have supported the hypothesis that higher SUA levels increase the risk of arterial stenosis. Most cross‐sectional and case‐control studies have suggested associations between SUA levels and subclinical atherosclerosis, by evaluating surrogates such as carotid plaque, carotid intima‐media thickness, coronary calcification and vascular stiffness.^5^, ^8^, ^26^ Few cohort studies have investigated the longitudinal association between SUA and atherosclerosis with a specific focus on middle‐aged adults. Several studies have indicated that while not the case in young adults, in whom high SUA levels are frequently accompany higher BMI, in middle‐aged adults, SUA may play a major role in predicting subclinical atherosclerosis.^12^, ^27^ Similarly, this study population was limited to middle‐aged and older adults, and the results show that SUA levels are associated with asymptomatic vascular stenosis independent of BMI. The potential mechanisms that might link uric acid levels and atherosclerosis are predominantly related to the ability of uric acid to induce oxidative stress and purine precursors may act as an enhancer of the xanthine oxidoreductase system,^28^ which might partly explain why the risk of vascular stenosis is raised even in subjects with SUA levels that are elevated but within the normal range. Moreover, renal impairment was a significant confounder in the association between SUA and atherosclerosis,^29^ thus all subjects with low eGFR (≤60 mL/min/1.73 m^2^) were excluded. The association between SUA and vascular stenosis was also analysed by eGFR stratification and further justified by a propensity score‐matched analysis conducted to better minimize the confounding effect of CKD. In all this context, our findings could be considered evidence supporting a role for SUA levels as a risk factor for subclinical atherosclerosis.

Consistent with the previous studies,^30^, ^31^ uric acid was found to be an independent risk factor for incidence of extracranial carotid atherosclerosis in both genders in this study. Yet, we found hyperuricaemia was associated with increased risk of ICAS only in men but not in women, completely opposite to two studies carried out in Korea and in the United States of America.^32^, ^33^ Higher SUA level was associated with increased risk of ICAS in middle‐aged Korea females but not in males.^33^ The ARIC Study also found the same gender specific effect on asymptomatic carotid atherosclerosis in white population.^32^ These inconsistences might have been attributed to the design (the Korean study was a cross‐sectional study while this study was a prospective cohort study), the ethnic difference and the measurement in determining atherosclerosis at different vascular territories. Moreover, SUA levels were higher in men than in women at all ages,^34^ which was also confirmed in this study. (Figure S2) This gender difference on SUA levels has been generally ascribed to estrogens that have the uricosuric effect in pre‐menopausal females^35^ and possibly to the overproduction and the impairment of renal clearance of uric acid in male individuals, especially in patients suffering from visceral fat obesity or metabolic syndrome.^36^ Moreover, the RCS results in this study indicated the range of SUA for increased risk in vascular stenosis was relatively lower in males than that in females. The gender‐specific effect of SUA on ICAS we report here might be partly due to the frequently higher levels of SUA in male individuals. In fact, when we selected a sample of male individuals with baseline SUA levels lower than 7.5 mg/dL, the significance of the association with ICAS and the gender difference was lost (*P* for interaction, 0.123). Therefore, it seems that there was threshold effect for SUA on ICAS, and this threshold may be more frequently reached by men than women.

We acknowledge several limitations in our study. First, we chose asymptomatic ICAS and ECAS as parameters to evaluate atherosclerosis. Causes of stenosis other than atherosclerosis (eg vasculitis, artery‐dissection, etc) were not evaluated in the study. Second, the correlation between elevated SUA levels and PAD was not analysed in this study because there were too few PAD events. Third, the risk for vascular stenosis may be attenuated by drugs lowering serum uric acid level, but the data about those participants who might have been treated with those drugs were not available. Additionally, ICAS was diagnosed by TCD, which is partly operator‐dependent and unable to accurately determine the extent of vascular stenosis. However, non‐invasive TCD is a safer, more accessible, and less expensive method of evaluating intracranial circulation than invasive catheter angiography.^37^

In this study, we found an association between elevated SUA levels and asymptomatic polyvascular stenosis, including ICAS, ECAS, and PAD. Chronic exposure to hyperuricaemia might accelerate the progression of ICAS, especially in men.

## CONFLICTS OF INTEREST

The authors confirm that there are no conflicts of interest.

## AUTHORS’ CONTRIBUTIONS

Cheng and Zhou designed the study and they equally contributed to the study. Song interpreted and analysed the data and prepared the report. Yao, Li, Wang, Cui commented on the manuscript draft and revised the report. Geng and Chen conducted the statistical analysis.
