# Supplementary material for: Longitudinal association between serum uric acid levels and multiterritorial atherosclerosis
Source: J Cell Mol Med. 2019 Jun 26;23(8):4970–9. doi: 10.1111/jcmm.14337 (PMC6652300; doi:10.1111/jcmm.14337)
Supplement: Supplementary file 1 [file JCMM-23-4970-s001.docx]

**Figure S1. Odd ratios with 95% CI for the onset of vascular stenosis in males and females due to hyperuricaemia and a 1-mg/dl elevation in SUA level in propensity-match analysis**


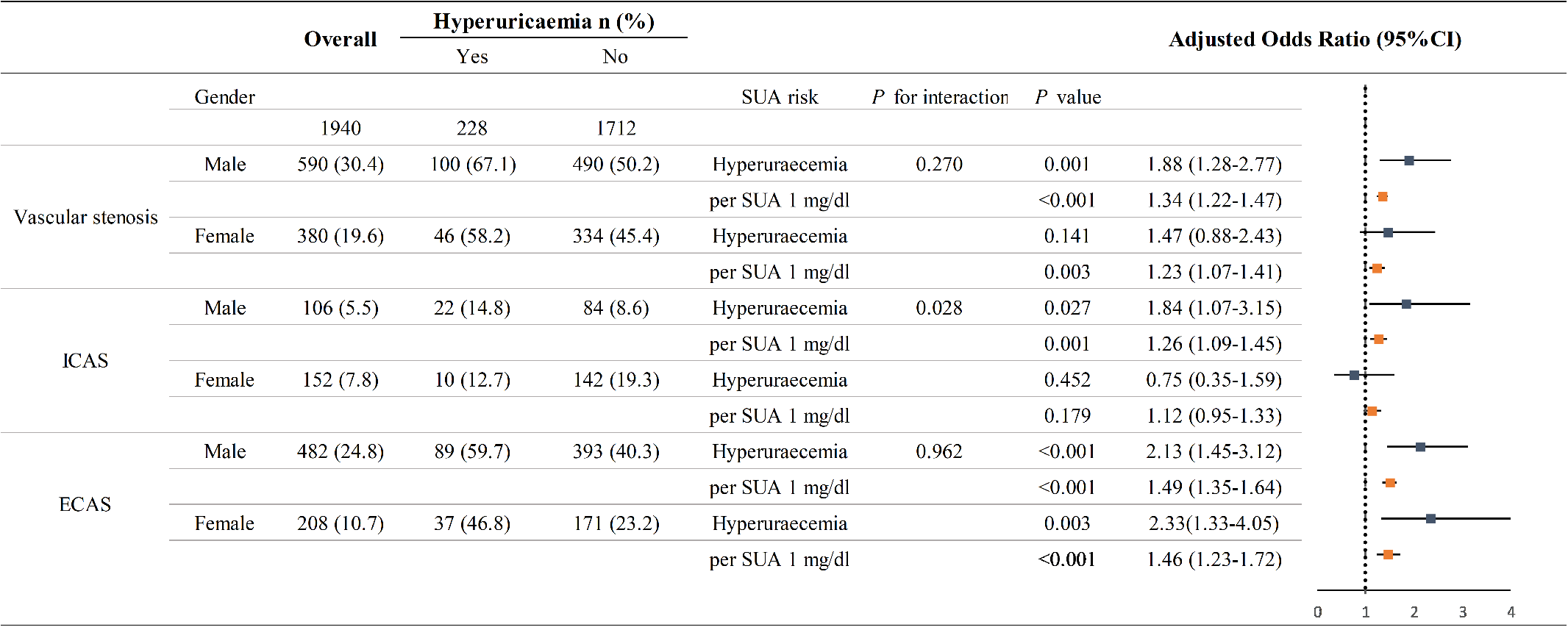


Fully adjusted for educational level, income, smoking, alcohol consumption, hypertension, hyperlipidaemia, diabetes mellitus, body mass index (BMI), C-reactive protein (CRP) and serum albumin (ALB). ICAS, intracranial artery stenosis; and ECAS, extracranial artery stenosis. Vascular stenosis was defined as the presence of ICAS or ECAS at ≥1 site.
