# Supplementary material for: Longitudinal association between serum uric acid levels and multiterritorial atherosclerosis
Source: J Cell Mol Med. 2019 Jun 26;23(8):4970–9. doi: 10.1111/jcmm.14337 (PMC6652300; doi:10.1111/jcmm.14337)
Supplement: Supplementary file 2 [file JCMM-23-4970-s002.docx]

Figure S2. SUA levels changing with age in males and females.


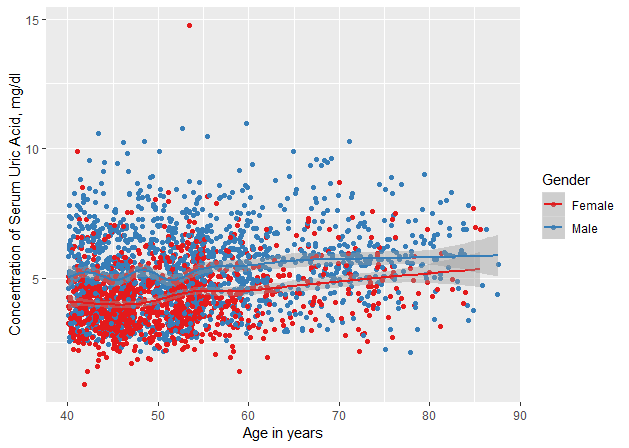


Males with higher SUA levels than females at all ages
