# Supplementary material for: Longitudinal association between serum uric acid levels and multiterritorial atherosclerosis
Source: J Cell Mol Med. 2019 Jun 26;23(8):4970–9. doi: 10.1111/jcmm.14337 (PMC6652300; doi:10.1111/jcmm.14337)
Supplement: Supplementary file 3 [file JCMM-23-4970-s003.docx]

| Characteristic | Vascular stenosis (n=970) | Non-Vascular stenosis (n=1670) | *P* value |
| --- | --- | --- | --- |
| **Age, mean (SD), y** | 57.9±11.78 | 50.57±8.28 | <0.001 |
| Male sex, n (%) | 1481(56) | 891(53.2) | <0.001 |
| BMI (SD), kg/m² | 24.97±3.32 | 24.81±3.12 | 0.211 |
| **Education level** |  |  | <0.001 |
| Primary school or low, n (%) | 243(9.2) | 127(7.6) |  |
| Middle or high school, n (%) | 1123(42.5) | 739(44.1) |  |
| College or above, n (%) | 1278(48.3) | 808(48.3) |  |
| **Income** |  |  | <0.001 |
| ≤500, n (%) | 27(1) | 14(0.8) |  |
| 500-1000, n (%) | 510(19.3) | 344(20.5) |  |
| 1000-3000, n (%) | 1789(67.7) | 1161(69.4) |  |
| ＞3000, n (%) | 318(12) | 155(9.3) |  |
| **Alcohol consumption** |  |  | 0.7 |
| Light, n (%) | 1787(67.6) | 1141(68.2) |  |
| Moderate, n (%) | 479(18.1) | 292(17.4) |  |
| Heavy, n (%) | 310(11.7) | 198(11.8) |  |
| **Smoking** |  |  | 0.057 |
| Never, n (%) | 1619(61.2) | 1071(64) |  |
| Once, n (%) | 88(3.3) | 49(2.9) |  |
| Currently, n (%) | 528(20) | 330(19.7) |  |
| **Physical activity** |  |  | <0.001 |
| Inactive, n (%) | 790(29.9) | 450(26.9) |  |
| Moderately active, n (%) | 1151(43.5) | 850(50.8) |  |
| Vigorously active, n (%) | 295(11.2) | 150(9) |  |
| **Hypertension, n (%)** | 1125(42.5) | 645(38.5) | <0.001 |
| **Diabetes mellitus, n (%)** | 257(9.7) | 130(7.8) | <0.001 |
| **Hyperlipidaemia, n (%)** | 1268(48) | 724(43.2) | <0.001 |
| TC (SD), mmol/L | 5.31±1.09 | 5.09±1.64 | 0.001 |
| TG (SD), mmol/L | 1.77±2 | 1.66±1.67 | 0.162 |
| HDL-C (SD), mmol/L | 1.43±0.46 | 1.39±0.45 | 0.04 |
| LDL-C (SD), mmol/L | 2.53±1.11 | 2.48±1.19 | 0.371 |
| **CRP (SD), mg/L** | 2.44±2.9 | 1.74±2.53 | <0.001 |
| **ALB (SD), g/L** | 46.25±3.04 | 47.2±20.39 | 0.254 |
| **SUA (SD), mg/dL** | 5.3±1.46 | 4.58±1.45 | <0.001 |
| **eGFR (SD), mL/min per 1.73 m²** | 101.78±20.44 | 98.95±23.41 | 0.002 |

**Table S1**. Univariate analyses on new vascular stenosis

Hyperuricaemia was defined as SUA ≥7.0 and ≥6.0 mg/dL in men and women, respectively.

BMI indicates body mass index; TG, triglyceride; TC, total cholesterol; HDL-C, high-density lipoprotein; LDL-C, low-density lipoprotein, CRP, C-reactive protein; ALB, Serum albumin; SUA serum uric acid and eGFR estimated glomerular filtration rate.
