# Supplementary material for: Longitudinal association between serum uric acid levels and multiterritorial atherosclerosis
Source: J Cell Mol Med. 2019 Jun 26;23(8):4970–9. doi: 10.1111/jcmm.14337 (PMC6652300; doi:10.1111/jcmm.14337)
Supplement: Supplementary file 4 [file JCMM-23-4970-s004.docx]

| Characteristic | Vascular stenosis (n=970) | Non-Vascular stenosis (n=970) | *P* value |
| --- | --- | --- | --- |
| **Age, mean (SD), y** | 57.9±11.78 | 54.22±8.72 | <0.001 |
| Male sex, n (%) | 590(60.8) | 535(55.2) | 0.013 |
| BMI (SD), kg/m² | 24.97±3.32 | 24.84±3.17 | 0.474 |
| **Education level** |  |  | 0.054 |
| Primary school or low, n (%) | 116(12.0) | 101(10.4) |  |
| Middle or high school, n (%) | 384(39.6) | 436(44.9) |  |
| College or above, n (%) | 470(48.5) | 433(44.6) |  |
| **Income** |  |  | <0.001 |
| ≤500, n (%) | 13 (1.3) | 6 (0.6) |  |
| 500-1000, n (%) | 166(17.1) | 182 (18.8) |  |
| 1000-3000, n (%) | 628(64.7) | 691(71.2) |  |
| ＞3000, n (%) | 163(16.8) | 91(9.4) |  |
| **Alcohol consumption** |  |  | 0.619 |
| Light, n (%) | 187(19.3) | 167(17.2) |  |
| Moderate, n (%) | 112(11.5) | 107(11.0) |  |
| Heavy, n (%) | 25(2.6) | 28(2.9) |  |
| **Smoking** |  |  | 0.128 |
| Never, n (%) | 548(69.8) | 595(74.4) |  |
| Once, n (%) | 39(5.0) | 34(4.2) |  |
| Currently, n (%) | 198(25.2) | 171(21.4) |  |
| **Physical activity** |  |  | <0.001 |
| Inactive, n (%) | 340(43.3) | 26.4(33.0) |  |
| Moderately active, n (%) | 301(38.3) | 436(54.5) |  |
| Vigorously active, n (%) | 145(18.4) | 100(12.5) |  |
| **Hypertension, n (%)** | 480(49.5) | 415(42.8) | 0.004 |
| **Diabetes mellitus, n (%)** | 127(13.1) | 98(10.1) | <0.001 |
| **Hyperlipidaemia, n (%)** | 544（56.1） | 446(40.6） | <0.001 |
| TC (SD), mmol/L | 5.31±1.09 | 5.19±2.05 | 0.12 |
| TG (SD), mmol/L | 1.77±2.00 | 1.66±1.86 | 0.242 |
| HDL-C (SD), mmol/L | 1.43±0.46 | 1.39±0.45 | 0.033 |
| LDL-C (SD), mmol/L | 2.53±1.11 | 1.49±0.83 | 0.452 |
| **CRP (SD), mg/L** | 2.44±2.9 | 1.95±2.67 | 0.003 |
| **ALB (SD), g/L** | 46.25±3.04 | 47.64±26.42 | 0.199 |
| **SUA (SD), mg/dL** | 5.3±1.46 | 4.69±1.46 | <0.001 |
| **eGFR (SD),** mL/min per 1.73 m² | 101.78±20.44 | 101.78±20.44 | 0.639 |

**Table S2. Baseline characteristics of participants with or without new vascular stenosis after propensity score match**

BMI indicates body mass index; TG, triglyceride; TC, total cholesterol; HDL-C, high-density lipoprotein; LDL-C, low-density lipoprotein, CRP, C-reactive protein; ALB, Serum albumin; SUA serum uric acid and eGFR estimated glomerular filtration rate.
