# Supplementary material for: Longitudinal association between serum uric acid levels and multiterritorial atherosclerosis
Source: J Cell Mol Med. 2019 Jun 26;23(8):4970–9. doi: 10.1111/jcmm.14337 (PMC6652300; doi:10.1111/jcmm.14337)
Supplement: Supplementary file 5 [file JCMM-23-4970-s005.docx]

**Table S3. The effect of hypouricaemia and hyperuricaemia on vascular stenosis**

|  |  | **Overall** | **Hyperuricaemia n (%)** | **Hypouricaemia n (%)** |  | | **Hypouricaemia** | |  | | **Hyperuricaemia** | | |
| --- | --- | --- | --- | --- | --- | --- | --- | --- | --- | --- | --- | --- | --- |
|  |  |  |  |  | ***P* for interaction** | **Adjusted Odds Ratio (95%CI)** | | ***P* value** | |  | | **Adjusted Odds Ratio (95%CI)** | ***P* value** |
|  | Gender | 2644 | 270 (10.21) | 12 (0.45) |  |  | |  | |  | |  |  |
| Vascular stenosis | Male | 590 (22.3) | 100 (56.8) | 0 | 0.171 | - | | - | |  | | 1.99 (1.39-2.85) | <0.001 |
|  | Female | 380 (14.4) | 46 (48.9) | 1 (8.3) |  | 0.30 (0.04-2.41) | | 0.259 | |  | | 1.53 (0.96-2.45) | 0.076 |
| ICAS | Male | 106 (4.0) | 22 (12.5) | 0 | 0.018 | - | | - | |  | | 2.02 (1.18-3.46) | 0.01 |
|  | Female | 152 (5.7) | 10 (10.6) | 0 |  | - | | - | |  | | 0.85 (0.41-1.76) | 0.669 |
| ECAS | Male | 482 (18.2) | 89 (50.6) | 0 | 0.948 | - | | - | |  | | 2.22 (1.54-3.21) | <0.001 |
|  | Female | 208 (7.9) | 37 (39.4) | 0 |  | - | | - | |  | | 2.34 (1.36-4.02) | 0.002 |

Fully adjusted for age, sex, educational level, income, smoking, alcohol consumption, hypertension, hyperlipidaemia, diabetes mellitus, body mass index (BMI), C-reactive protein (CRP), serum albumin (ALB) and estimated glomerular filtration rate (eGFR). ICAS, intracranial artery stenosis; and ECAS, extracranial artery stenosis. Vascular stenosis was defined as the presence of ICAS or ECAS at ≥1 site.
